# Compliance with the World Health Organization’s 2016 prenatal care contact recommendation reduces the incidence rate of adverse birth outcomes among pregnant women in northern Ghana

**DOI:** 10.1371/journal.pone.0285621

**Published:** 2023-06-08

**Authors:** Leticia Achangebe Akum, Eunice Amina Offei, Mary Rachael Kpordoxah, Daudi Yeboah, Abdul-Nasir Issah, Michael Boah

**Affiliations:** 1 Department of Population and Reproductive Health, School of Public Health, University for Development Studies, Tamale, Ghana; 2 Department of Midwifery and Women’s Health, School of Nursing and Midwifery, University for Development Studies, Tamale, Ghana; 3 Department of Global and International Health, School of Public Health, University for Development Studies, Tamale, Ghana; 4 Department of Epidemiology, Biostatistics, and Disease Control, School of Public Health, University for Development Studies, Tamale, Ghana; 5 Department of Health Services, Policy, Planning, Management and Economics, School of Public Health, University for Development Studies, Tamale, Ghana; Bangabandhu Sheikh Mujib Medical University (BSMMU), BANGLADESH

## Abstract

**Background:**

Children born with adverse birth outcomes (ABOs) have a greater risk of mortality, stunting, and poor cognitive development. In 2016, the World Health Organization (WHO) recommended at least eight antenatal care (ANC) contacts before delivery for a healthy mother and baby. We examined the association between compliance with this recommendation and the risk of ABOs, such as low birthweight (LBW) and preterm birth (PTB), in the Tamale Metropolitan Area of the northern region of Ghana.

**Methods:**

We conducted a cross-sectional study in the Tamale Metropolis of the northern region of Ghana. We analysed a systematic random sample of 402 postnatal women aged 15–49 drawn from five public health facilities. We gathered information electronically on their birth outcomes, specifically their birthweight and gestation at delivery, using a structured questionnaire. Information on women’s background characteristics, including the number of ANC contacts made before delivery, was also collected. The association between the number of ANC contacts and ABOs was investigated using regression models.

**Results:**

We found that 37.6% (95% CI: 32.9, 42.4) of our sample had at least eight ANC contacts before delivery. We estimated that 18.9% of babies were born prematurely and 9.0% were born LBW. ABOs were found in 22.9% (95% CI: 19.0, 27.3) of babies. A minimum of eight ANC contacts before delivery reduced the risk of ABOs (adjusted IRR = 0.43; 95% CI: 0.25, 0.73), PTB (AOR = 0.28; 95% CI: 0.14, 0.58), and LBW (AOR = 0.36; 95% CI: 0.14, 0.91).

**Conclusion:**

In the current study’s setting, about a quarter of newborns have ABOs, jeopardising their survival, health, and development. Compliance with at least eight ANC contacts prior to birth was associated with a reduced incidence rate ratio of ABOs. However, less than four out of every ten pregnant women make at least eight ANC contacts before delivery. Efforts are needed to increase coverage of eight contacts among pregnant women before delivery to reduce the risk of ABOs in the study setting.

## Introduction

Adverse birth outcomes (ABOs), such as preterm birth (PTB) and low birthweight (LBW), are substantial public health problems affecting many low- and middle-income countries (LMICs), largely in Asia and sub-Saharan Africa (SSA). According to international classifications, a baby is preterm if it is born before 37 completed weeks of gestation but after 28 weeks of gestation, and it has a low birthweight if its birth weight is below 2500 g [1]. Globally, approximately 11% of live newborns are born prematurely each year, which translates into 14.8 million preterm births annually, with nearly 81% occurring in Asia (53.9%) and SSA (25%) [2]. Similarly, about 15% of neonates are born with a LBW, translating into 20.5 million live births each year, with the majority of these births occurring in Asia (48%) and SSA (24%) [3].

Babies born prematurely or with a low birthweight are at a greater risk of morbidity and mortality due to the complications that come with them [4,5]. PTB and LBW babies also suffer both short- and long-term developmental challenges. Poor cognitive development is more common among children born preterm than those born at term [6]. LBW newborns were also more likely to be stunted, wasted, and underweight in early childhood [7]. The causes of preterm births are not clearly understood. However, it has been established that the risk of PTB is increased among women who have hypertensive disorders during pregnancy, including preeclampsia and eclampsia [8]. Rahman and his colleagues [9] also found that anaemia during pregnancy also increased the risk of ABOs. Antenatal care (ANC) offers an opportunity for healthcare professionals to screen for these pregnancy-related risk factors and provide cost-effective interventions to avert ABOs in women [10]. Having at least one ANC visit during pregnancy is associated with a 3.82% points reduction in the probability of giving birth to a LBW neonate among women from LMICs [11]. Unfortunately, a significant proportion of pregnant women in SSA do not attend ANC, attend late, or do not attend frequently enough [12,13]. It is established that in countries with high maternal mortality, slightly over half (55.6%) of pregnant women make at least four ANC visits before delivery [14].

In 2016, the WHO revised its earlier recommendation on the number of ANC visits from a minimum of four to eight contacts for a positive pregnancy experience [15]. This became necessary after several considerations, including the fact that the old model is associated with more perinatal deaths than ANC models that include at least eight contacts between pregnant women and the health care provider [16]. Evidence available from nationally representative data collected after 2016 shows that coverage of eight or more contacts among pregnant women is as low as 1% in some African countries, such as Senegal, Uganda, and Zambia, and 43% in Ghana [17]. In other words, it appears that this recommendation has worsened the ANC coverage indicator in settings where coverage of four visits is already low. Non-compliance with the minimum of eight ANC contacts among women in SSA has been associated with individual and community factors, including maternal age, health insurance coverage, parity, household socioeconomic status, place of residence, and community-level literacy [18,19].

Despite the fact that Ghana has implemented the new ANC contact recommendation, it is unclear how compliance with this relates to ABOs among pregnant women; there is a lack of literature on the relationship between compliance with the new recommendation on frequency of ANC contacts and ABOs in Ghana. The northern region of Ghana is of particular concern because, according to national reports, more women in this region are less likely to use prenatal care when compared to the other administrative regions [20]. The existing studies, on the other hand, have associated maternal age, parity, and pregnancy-related complications, such as hypertension and premature rapture of membranes, with ABOs in Ghana, including PTB and LBW [21,22].

The current study examined the association between compliance with the new recommendation for frequency of ANC contacts and the risk of ABOs such as PTB and LBW among reproductive women in the Tamale Metropolitan Area of the northern region of Ghana.

## Materials and methods

### Study design and setting

A retrospective, cross-sectional study was conducted at five public health facilities in the northern Ghanaian city of Tamale. The northern region of Ghana is divided into sixteen districts, and Tamale serves as the capital of one of those districts: the Tamale Metropolis. It is bounded to the north by Savelugu Municipality, to the south by East Gonja Municipality, to the south-west by Central Gonja District, to the east by Yendi Municipal Assembly, and to the west by Tolon District [23]. The Metropolis serves a population of 374,744 people with its public health infrastructure, which consists of three hospitals, one of which is a teaching hospital, eight health centres, and eighteen Community-based Health Planning and Services (CHPS) compounds. The Metropolis’ urban areas are home to more than 80% of the region’s population.

### Sample and sample size

Women aged 15–59 years who had given birth within the previous twelve months were included in the study. The sample size was calculated using the Taro Yamane formula as follows:

$$n=\frac{N}{\left(1+Ne^{2}\right)}$$ (1)

where “n” is the desired sample size, “N” is the cumulative expected number of births at the targeted facilities, and “e” is the margin of error (5%) allowed in the study. In order to achieve the objective of the current study, we estimated that a sample size of 402 was required, which includes a 10% for non-response. The study’s participants were partnered women who had given birth to a single child, had young children (under 12 months), received postpartum or child welfare care services, had ANC records, and agreed to participate.

### Selection of facilities and respondents

For the purpose of this research, a multisampling approach was used to select both the public health facilities and the respondents. In the first step of the process, the hospitals and clinics were chosen. Because they offered ANC and delivery services to a sizeable number of reproductive women in the region, the Tamale Central Hospital and the Tamale West Hospital were chosen on purpose as the two secondary public health facilities to conduct the study at. In addition to the two secondary-level facilities, three health centres—the Datoyili health centre, the Bilpiela health centre, and the Vittin Reproductive and Child Health centre—were chosen at random from the eight that were located in the Metropolis. To ensure that each facility received an adequate representation of the study’s sample, the proportion-to-size technique was used.

During the second step of the sampling procedure, respondents were selected using a systematic random sampling technique. At each of the selected health facilities, the postnatal register was used to compile a list of partnered women who had given birth within the previous twelve months prior to the survey. Afterwards, a sampling interval was determined with the help of the sampling frame by dividing the total number of women who were qualified to participate in the study by the minimum required sample size for the facility. Starting with the first ten names in the sampling frame, a random participant was selected. This served as the starting point for the process. After that, respondents were selected using the sampling interval until the target sample size was reached.

### Data collection and methods

The data for this study were collected electronically using the Kobo Collect application on Android-powered mobile phones. A total of forty women, which represented approximately 10% of the overall sample size, were recruited from a health facility outside of the study setting to pilot test the data collection tool. To ensure clarity, we rephrased any questions that were not easily understood by the respondents. The data collection tool covered various aspects, including socioeconomic status, obstetric history, and maternal health. Additionally, information on gestational age, haemoglobin levels, and birthweight of the baby was obtained by reviewing the ANC record book. The data were collected by trained research assistants with nursing degrees who were supervised by the research team. Data collection commenced in June 2022 and ended in August the same year.

### Variables

#### Adverse birth outcomes

We defined ABOs, the outcome variable, using PTB and LBW. According to the International Statistical Classification of Diseases—10^th^ revision (ICD-10), 5^th^ edition, a baby is preterm if it is born before 37 completed weeks of gestation but after 28 weeks of gestation and has a low birth weight, if it weighs less than 2500 g at birth [1]. A woman is said to have experienced an ABO in the current study if she delivered preterm, a low birthweight baby, or both.

#### Explanatory variables

The main explanatory variable in the study is the number of prenatal care contacts made before delivery. We used the 2016 WHO recommendations to classify the respondents into two groups: fewer than eight and at least eight ANC contacts [15]. There are several factors that are well-established in the literature to impact birth outcomes [24–27]. As a result, factors, including sex of the child, maternal age, education, and employment status, anaemia during the first, second, and both first and second trimesters, and exposure to psychological intimate partner violence (IPV), were considered as potential confounding factors in the current study. Exposure to any acts of psychological IPV was determined by asking women “whether their partner ever humiliated, threatened with harm, or insulted, or made them feel bad” during their most recent pregnancy. A dichotomous variable was created, with “yes” representing women who reported experiencing any act of psychological IPV during their most recent pregnancy and “no” representing women who did not experience any acts of psychological IPV. Gravidity of the woman was used in a continuous form.

#### Data analysis

We first estimated the prevalence of LBW, PTB, and ABOs among the study sample. Then we examined the distribution of ABOs across the explanatory variables using the chi-square (χ^2^) or Fisher’s test, where appropriate. Both simple and multiple binary regression models were used to quantify the association between the number of prenatal contacts and the risk of LBW and PTB. Lastly, we performed Poisson regression analysis to evaluate the association between the number of prenatal contacts and the number of ABOs experienced, while adjusting for potential confounding by child and maternal-level factors. We checked for multicollinearity of the explanatory variables before fitting them into the Poisson model; none was found to be collinear. We reported the results of the Poisson regression in the Incident Rate Ratio (IRR) by exponentiating the Poisson coefficients. Statistical significance was set at a probability value (p-value) of less than 0.05. The chi-square goodness-of-fit test was used to assess the fit of the Poisson regression model. The goodness-of-fit Chi-square test was not statistically significant (Pearson goodness-of-fit = 393.9346; Prob > chi2 (389) = 0.421). As a result, we concluded that our Poisson model fit reasonably well. All the statistical analyses were performed using Stata/IC 15.0 (StataCorp LLC, College Station, USA). The “vce (robust)” option was used in our Poisson parameter estimations to control for potential variation of the underlying assumptions.

#### Ethics

The Ghana Health Service Ethic Review Committee (ERC) granted approval for the current study (GHS-ERC 033/11/22). Permission was sought from the various heads of the health institutions before conducting the study. Verbal and written informed consent was obtained from all participants before being interviewed. All participants’ data were anonymized before analysis. The study was conducted according to the principles guiding human research subjects in the Helsinki Declaration.

## Results

### Prevalence of adverse birth outcomes among women in the current study

**Fig 1:**
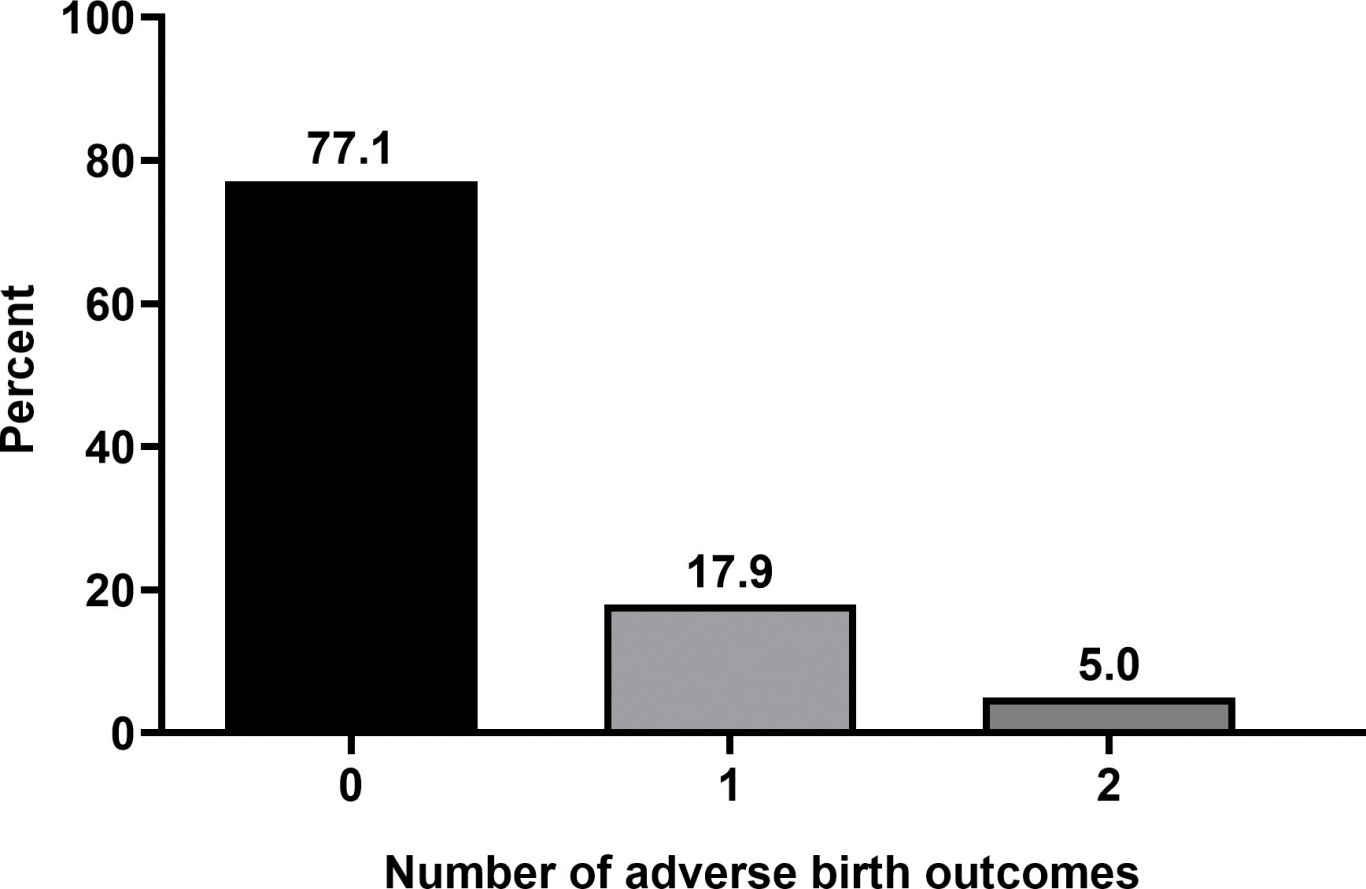
Number of adverse birth outcomes (ABOs) among the study sample (N = 402).

We estimated that 18.9% (95% CI: 15.4, 23.1) of the 402 babies were born preterm and 9.0% (95% CI: 6.5, 12.2) were born with a low birthweight. In total, 77.1% of the babies were born with no ABO, 22.9% (95% CI: 19.0, 27.3) had at least one type of ABO, 17.9% had only one type of ABO, and 5.0% had two types of ABOs (Fig 1).

### Distribution of the number of adverse birth outcomes by the number of antenatal care contacts and the various explanatory variables in the current study

Table 1 presents the distribution of the number of ABOs across the explanatory variables. The results showed that 37.6% (95% CI: 32.9, 42.4) of the total sample had at least eight ANC contacts prior to delivery. A higher proportion (88.7%) of women who made at least eight ANC contacts before delivery did not experience any ABO compared to those who made fewer than eight contacts (70.1%). At the same time, a significantly high proportion of those who made fewer than eight ANC contacts prior to delivery experienced one (23.1%) or two (6.8%) ABOs compared to those who made at least eight follow-up contacts (9.3% and 2.0%, respectively). There were also marked differences in the number of ABOs by sex of the child, maternal age, employment, malaria infection during recent pregnancy, anaemia in the first trimester, anaemia in both the first and second trimesters, and exposure to psychological IPV during pregnancy. ABOs were less common in women aged 35–49 years (90.0%), employed (84.3%), not infected with malaria during their recent pregnancy (75.4%), not anaemic in the first trimester (80.0%) and both the first and second trimesters (77.8%), and women who did not experience psychological IPV during their recent pregnancy (79.8%).

**Table 1:** Distribution of the number of adverse birth outcomes across the explanatory variables (N = 402).

Variable	n (%)	Percent distribution of the number of ABOs			p-value
		0 (No ABO)	1 (Either LBW or PTB)	2 (Both LBW and PTB)	
Number of ANC contacts					<0.001^a^
1–7	251(62.4)	70.1	23.1	6.8	
8 or more	151(37.6)	88.7	9.3	2.0	
Child-level factors					
Sex of child					0.005
Female	103(25.6)	68.9	20.4	10.7	
Male	299(74.4)	79.9	17.1	3.0	
Maternal factors					
Age group					<0.001^a^
15–24	47(11.7)	55.3	27.7	17.0	
25–34	235(58.5)	74.9	20.8	4.3	
35–49	120(29.9)	90.0	8.3	1.7	
Received formal education					0.919
No	258(64.2)	77.5	17.8	4.7	
Yes	144(35.8)	76.4	18.1	5.5	
Employed					<0.001
No	96(23.9)	54.2	35.4	10.4	
Yes	306(76.1)	84.3	12.4	3.3	
Religion					0.467^a^
Christian	17(4.2)	70.6	29.4	0.0	
Islam	385(95.8)	77.4	17.4	5.2	
Gravidity					
Malaria infection during pregnancy					0.035
No	285(70.9)	75.4	17.9	6.7	
Yes	117(29.1)	81.2	18.0	0.8	
Anaemic during 1^st^ trimester pregnancy					0.018
No	320(79.6)	80.0	15.3	4.7	
Yes	82(20.4)	65.9	28.0	6.1	
Anaemic during 2^nd^ trimester pregnancy					0.650
No	273(67.9)	76.9	18.7	4.4	
Yes	129(32.1)	77.5	16.3	6.2	
Anaemic during both 1^st^ and 2^nd^ trimesters of pregnancy					0.030^a^
No	392(97.5)	77.8	17.6	4.6	
Yes	10(2.5)	50.0	30.0	20.0	
Exposed to psychological IPV during pregnancy					<0.001^a^
No	262(65.2)	79.8	18.7	1.5	
Yes	140(34.8)	72.2	16.4	11.4	

^a^Fisher’s exact test p-values: ANC: Antenatal care; APOs: Adverse pregnancy outcomes; IPV: Intimate partner violence.

### Analyses of the association between the number of prenatal care contacts and low birthweight and preterm birth by logistic regression

Table 2 presents the results of the binary logistic regression analyses. Both the unadjusted and adjusted logistic regression models showed that the number of ANC contacts was significantly associated with LBW and PTB. According to the results, women who had at least eight prenatal contacts before giving birth had a lower risk of delivering LBW or prematurely. In the adjusted analyses, women with at least eight ANC contacts were 36% less likely to deliver low birthweight babies (AOR = 0.36; 95% CI: 0.14, 0.91) and 28% less likely to deliver preterm babies (AOR = 0.28; 95% CI: 0.14, 0.58). Male children had lower odds of being born LBW than female children, but the odds of being born LBW were about three times higher among women who were anaemic in the first trimester (AOR = 3.34; 95% CI: 1.27, 8.75) and those who were exposed to psychological IPV during pregnancy (AOR = 2.73; 95% CI: 1.26, 5.90) than those who were not. Employed women, compared to unemployed women, had reduced odds of delivering preterm (AOR = 0.35; 95% CI: 0.19, 0.65). Similarly, an increase in the gravidity reduced the odds of delivering preterm babies by 55% (AOR = 0.55; 95% CI: 0.39, 0.77).

**Table 2:** Logistic regression analyses of the association between the number of prenatal care contacts and low birthweight and preterm birth (N = 402).

Variable	Low birthweight		Preterm birth	
	OR [95% CI]	AOR [95% CI]	OR [95% CI]	AOR [95% CI]
Number of ANC contacts				
1–7	1.00	1.00	1.00	1.00
8 or more	0.37*[0.16,0.87]	0.36* [0.14,0.91]	0.28***[0.15,0.53]	0.28***[0.14,0.58]
Child-level factors				
Sex of child				
Female		1.00		1.00
Male		0.24***[0.11,0.50]		0.71 [0.37,1.38]
Maternal factors				
Age group				
15–24		1.00		1.00
25–34		0.35 [0.12,1.04]		0.61 [0.28,1.37]
35–49		0.77 [0.16,3.65]		0.33 [0.08,1.28]
Received formal education				
No		1.00		1.00
Yes		0.84 [0.36,1.93]		0.92 [0.48,1.79]
Employed				
No		1.00		1.00
Yes		1.03 [0.43,2.48]		0.35***[0.19,0.65]
Religion				
Christian		1.00		1.00
Islam		1.03 [0.12,9.17]		0.41 [0.11,1.52]
Gravidity		0.72 [0.48,1.09]		0.55***[0.39,0.77]
Malaria infection during pregnancy				
No		1.00		1.00
Yes		0.97 [0.40,2.35]		0.76 [0.37,1.59]
Anaemic during 1^st^ trimester of pregnancy				
No		1.00		1.00
Yes		3.34*[1.27,8.75]		1.47 [0.65,3.31]
Anaemic during 2^nd^ trimester of pregnancy				
No		1.00		1.00
Yes		1.39 [0.53,3.62]		0.97 [0.49,1.94]
Anaemic during both 1^st^ and 2^nd^ trimesters of pregnancy				
No		1.00		1.00
Yes		0.93 [0.12,7.21]		3.56 [0.57,22.30]
Exposed to psychological IPV during pregnancy				
No		1.00		1.00
Yes		2.73*[1.26,5.90]		1.26 [0.69,2.30]

ANC: Antenatal care; AOR: Adjusted odds ratio; IPV: Intimate partner violence; OR: Odds ratio.

* *p* < 0.05

** *p* < 0.01

*** *p* < 0.001.

### Poisson regression analysis of the association between making at least eight ANC contacts and the risk of adverse birth outcomes

The Poisson regression models examined the association between the number of ANC contacts and the number of ABOs (Table 3). The bivariate result showed that the two variables were significantly associated. We found that women who made at least eight contacts prior to delivery had a low incident rate ratio of ABOs compared to those who made fewer than eight contacts (Crude IRR = 0.36; 95% CI: 0.22, 061). The association remained statistically significant after controlling for potential confounding. The adjusted model identified that if a woman made at least eight ANC contacts before delivery, then the incident rate ratio for the number of ABOs would decrease by 0.42 (adjusted IRR = 0.43; 95% CI: 0.25, 0.73). In addition, among the covariates, we found that the sex of the child, being employed, gravidity, and being anaemic in the first trimester predicted the number of ABOs. Male newborns compared to females had a rate 0.61 times lower for ABOs (adjusted IRR = 0.61; 95% CI: 0.43, 0.87). In addition, the rate ratio for the number of ABOs would be expected to decrease by 0.65 (adjusted IRR = 0.65; 95% CI: 0.44, 0.95) if the woman is employed compared to if she is not. Similarly, with an increase in the gravidity of a pregnant woman by one pregnancy, her rate ratio for the number of ABOs would be expected to decrease by a factor of 0.69 (adjusted IRR = 0.69; 95% CI: 0.54, 0.88). In contrast, women who are anaemic in the first trimester compared to those who are not are expected to have rate ratio that is about two times greater for the number of ABOs (adjusted IRR = 1.54; 95% CI: 1.02, 2.32). Higher incident rate ratios were found among women who were anaemic in the second trimester and both the first and second trimesters, as well as those exposed to psychological IPV during pregnancy, although the relationships were statistically insignificant (p > 0.05).

**Table 3:** Poisson regression analyses of the association between making at least eight antenatal care contacts and the risk of adverse birth outcomes (N = 402).

Variable	Adverse birth outcomes (ABOs)	
	Crude IRR [95% CI]	Adjusted IRR [95% CI]
Number of ANC contacts		
1–7	1.00	1.00
8 or more	0.36***[0.22,0.61]	0.42** [0.25,0.73]
Child-level factors		
Sex of child		
Female		1.00
Male		0.61**[0.43,0.87]
Maternal factors		
Age group		
15–24		1.00
25–34		0.67 [0.45,1.00]
35–49		0.64 [0.27,1.51]
Received formal education		
No		1.00
Yes		0.95 [0.66,1.36]
Employed		
No		1.00
Yes		0.65*[0.44,0.95]
Religion		
Christian		1.00
Islam		0.64 [0.32,1.27]
Gravidity		0.69** [0.54,0.88]
Malaria infection during pregnancy		
No		1.00
Yes		0.92 [0.60,1.39]
Anaemic during 1^st^ trimester of pregnancy		
No		1.00
Yes		1.54*[1.02,2.32]
Anaemic during 2^nd^ trimester of pregnancy		
No		1.00
Yes		1.03 [0.68,1.56]
Anaemic during both 1^st^ and 2^nd^ trimesters of pregnancy		
No		1.00
Yes		1.59 [0.53,4.78]
Exposed to psychological IPV during pregnancy		
No		1.00
Yes		1.37 [0.96,1.94]
Final model fitness test		
Pearson goodness-of-fit		393.935
Prob > chi2 (389)		0.421

ANC: Antenatal care; IPV: Intimate partner violence; IRR: Incident rate ratio.

* *p* < 0.05

** *p* < 0.01

*** *p* < 0.001.

## Discussion

We examined the association between compliance with the new WHO recommendation on frequency of ANC contacts and the risk of ABOs, such as LBW and PTB, in the Tamale Metropolitan Area of the northern region of Ghana. We cautiously claim that this is the first study on this topic in this setting. According to our study, slightly over one-third (38%) of pregnant women in the study setting made the minimum of eight ANC contacts, which is lower than the national average but higher than the regional average. In Ghana, four out of every ten reproductive women (42%) have at least eight ANC contacts during pregnancy, with about a quarter of those (24.4%) occurring in the northern region, the third lowest after the upper west (20.1%) and Volta (23.1%) regions [28]. Differences in study designs may explain the observed differences in rates.

We found that, in total, about a quarter of newborns were delivered with some form of ABO. Specifically, approximately 19% were born prematurely, and 9% were LBW. Furthermore, 5% were born both prematurely and with LBW. The prevalence of LBW in this study is lower than the 14% reported in the upper east region [29], but higher than the 8% in the upper west region [30]. The prevalence is also lower than the global average of nearly 15% [31]. On the other hand, the rate of premature delivery in the current study is similar to what has been reported (18.9%) in a tertiary hospital in southern Ghana [22]. A facility-based cross-sectional study in northeast Ethiopia [27] reported that approximately 32% of deliveries resulted in ABOs defined by LBW, PTB, or stillbirth, which is higher than the rate in the current study. An analysis of DHS data from ten SSA countries reported a pooled prevalence rate of ABOs of about 30%, defined by the presence of one or more of the following: LBW, macrosomia, PTB, and stillbirth [32]. Heterogeneity between the results from Ethiopia and the current study may be explained in part by contextual factors and different ABOs measures. Indeed, we have observed that studies [26,27,32,33] that measured ABOs using at least three outcomes reported relatively higher rates.

Preventive measures including deworming, iron and folic acid supplementation, and intermittent preventive treatment of malaria in pregnancy (IPTp) given to women at ANC are known to reduce the risk of ABOs, including LBW and PTB [34,35]. Furthermore, antenatal nutritional education on increased energy and protein intake among pregnant women reduced the risk of LBW and PTB by 96% and 54%, respectively [36]. An assessment of 193 surveys in 69 low- and middle-income countries has associated ANC with improved birth outcomes. According to the study, at least one ANC visit is associated with a 3.8% point reduction in the probability of giving birth to a LBW baby, and having at least four ANC visits with at least one of them being with a skilled provider reduced the probability by an additional 2.8% [11]. From their analysis of demographic and health data from four (ASEAN) countries, Arsyi and colleagues reported that more pregnant women in Indonesia, Cambodia, and Myanmar who made fewer ANC contacts and did not receive the full components of prenatal care interventions before delivery had LBW babies compared to their counterparts [37]. A study in a teaching hospital in southern Ghana found that an increase in the number of ANC contacts reduced the odds of PTB [21].

Consistent with these observations, we found significant associations between compliance with the new ANC contact recommendation and the risk of ABOs. Our findings showed that women who made a minimum of eight ANC contacts before delivery had a lower risk of ABOs. Specifically, a reduction in the risk of low birthweight and preterm delivery by 36% and 28%, respectively, as shown by our adjusted binary regression models, and a reduction in the incident rate ratio of ABOs by a factor of 0.42, as revealed by our Poisson regression model. The 2016 WHO ANC guidelines propose that the first contact should take place in the first trimester, followed by two contacts in the second trimester, and five contacts in the third trimester [15]. The guidelines also clearly outline the interventions and the contact (s) that pregnant women should receive in order to have a healthy mother and baby. As a result, pregnant women who make fewer than eight contacts before delivery may miss the opportunity to receive some essential ANC interventions that are expected to reduce the risk of ABOs before delivery, as demonstrated by a study in Cameroon [38]. This, in part, may explain the high risk of ABOs among women who made fewer than the eight contacts in the current study.

The direction of the association between sex of the child and the risk of ABOs in SSA appears to be inconsistent across different studies, and the results vary depending on the specific outcomes and populations studied. For instance, in contrast to the present study, a pooled analysis of DHS data from Angola, Congo, Cote d’Ivoire, Gambia, Lesotho, Liberia, Madagascar, Nigeria, Rwanda, and Togo indicated that female newborns had a lower risk of adverse birth outcomes [32]. Adverse birth outcomes in the said study were defined as LBW, stillbirth, macrosomia, and PTB. However, in another study examining the prevalence of low birth weight and its associated factors in 35 countries in SSA, female newborns were more susceptible to LBW compared to male newborns [39]. Similar findings were reported in Ghana with regards to female sex and low birth weight [29,40]. Furthermore, a recent analysis of rates and risk factors for preterm birth and low birth weight in six low- and low-middle-income countries revealed that female newborns in African sites were more likely to be born preterm [41]. More research is needed to better understand the association between the sex of the child and adverse birth outcomes in the SSA region.

Our findings indicate that employed women are less likely to experience ABOs compared to unemployed women. This may be attributed to the greater access to healthcare services and higher socioeconomic status of employed women, which enables them to avail of better prenatal care, nutritious food, and a safe living environment [42,43]. Furthermore, in the present study, the risk of ABOs decreased with an increase in gravidity, which is consistent with existing research in SSA [44,45]. However, nulligravid women and those with high gravidity are both at elevated risk for ABOs due to a lack of prior pregnancy experience and complications related to uterine overdistension or maternal depletion syndrome, respectively. While we acknowledge that high gravida may increase the risk of ABOs, the age at first pregnancy may also play a significant role in determining the magnitude of this risk [46]. It is plausible that women who have had multiple pregnancies may have a better understanding of prenatal care and more experienced reproductive systems, leading to better outcomes for newborns.

We found that anaemia during pregnancy, particularly in the first trimester, increased the risk of ABOs. Anaemia during pregnancy can lead to inadequate oxygen supply to the foetus, which can result in LBW and PTB. Iron-deficiency anaemia is the most common form of anaemia during pregnancy, and it can have a negative impact on foetal growth and development [47]. According to a study, about a quarter of women with anaemia during the first trimester had their condition rectified in the later stages of pregnancy [48]. This suggests that a substantial proportion of women with anemia during the initial trimester tend to continue experiencing the condition throughout the subsequent stages of pregnancy. By addressing anemia early in pregnancy, the risk of adverse foetal outcomes can be reduced, ultimately leading to improved child health outcomes.

### Policy implications of the current study’s findings

The findings of the current study have serious implications for policy within the study setting. First, a significant percentage of children in the study setting are born with some ABOs. These children are more likely to die early within the first month of their lives or suffer long-term consequences such as undernutrition and poor cognitive development [4,6,49,50]. Those who survive are more likely to suffer from other chronic health conditions in adulthood, including diabetes and cardiovascular diseases [51,52]. However, there is a window of opportunity to avert these adverse consequences. Although we did not examine the ANC interventions received by the women or at what contact a particular intervention was received, the findings demonstrated that when women make at least eight contacts before delivery, they are less likely to deliver prematurely and have babies with LBW. This finding reemphasizes the importance of adequate ANC as an ingredient for a healthy pregnancy, which helps to identify and manage the conditions that cause ABOs for babies to stay alive and thrive. However, the majority of pregnant women in the study area are not able to make a minimum of eight contacts before delivery, which may limit their opportunity to receive the essential prenatal interventions to avert ABOs. As a result, more efforts are needed to increase the proportion of women who are able to make at least eight contacts prior to delivery to reduce the risk of ABOs, such as PTB and LBW.

### Strengths and limitations of the current study

One of the limitations of the current study is that it used a cross-sectional design among reproductive women who used public health facilities in the study setting. As a result, we are limited in our ability to draw causal inferences and generalisation to all women in the study setting, including those who do not use public health facilities and those who are not of reproductive age. Another limitation worth mentioning is that we did not include all factors with a known association with ABOs as potential confounding factors. Furthermore, there are many ABOs, including stillbirths, miscarriages, macrosomia, and post-partum hemorrhages; however, we examined only LBW and PTB. Despite these limitations, we used robust statistical analysis in our estimations, and our final model was a good fit for achieving the main objective of the current study.

## Conclusions

In the current study’s setting, about a quarter of newborns are born prematurely and/or with LBW, jeopardising their survival, health, and development. Compliance with a minimum of eight ANC contacts before delivery was associated with a reduced incidence rate ratio of ABOs. However, less than four in every ten pregnant women are able to make at least eight ANC contacts before delivery. Efforts are needed to increase coverage of at least eight contacts among pregnant women before delivery to reduce the risk of ABOs in the study setting.

## Abbreviations

ABOs: Adverse births outcomes
ANC: Antenatal care
AOR: Adjusted odds ratio
CHPS: Community-based Health Planning and Services
CI: Confidence interval
ERC: Ethics review committee
IPTp: Intermittent preventive treatment of malaria in pregnancy
IPV: Intimate partner violence
IRR: Incidence rate ratio
LBW: low birthweight
LMICs: low- and middle-income countries
PTB: Preterm birth
SSA: sub-Saharan Africa
WHO: World Health Organization
